# A case report of Paliperidone palmitate-induced anaphylaxis

**DOI:** 10.1192/j.eurpsy.2024.1450

**Published:** 2024-08-27

**Authors:** S. Pereira, J. Pais

**Affiliations:** Department of Psychiatry and Mental Health, Centro Hospitalar do Tâmega e Sousa, Penafiel, Portugal

## Abstract

**Introduction:**

Paliperidone Palmitate (PP) is an atypical antipsychotic, approved by the FDA for acute and maintenance treatment of schizophrenia and schizoaffective disorder.

It has a relatively safety profile, and reported cases of paliperidone palmitate-induced angioedema or anaphylaxis are uncommon.

**Objectives:**

We intend to present a case of paliperidone palmitate-induced anaphylaxis to alert clinicians regarding this rare, but possible complication.

**Methods:**

Non-systematic review of the literature and report of a case study.

**Results:**

Long-acting injectable Paliperidone Palmitate (LAIPP) is a safe and effective alternative to oral Paliperidone, with less incidence of disease relapse related to medication non-compliance.

Substance use disorder (SUD) is highly prevalent in first-episode psychosis (FEP), and it is associated to decreased treatment compliance, which impairs the outcomes of these patients. Therefore, several authors have been recommended long-acting injectable antipsychotics (LAI-AP), such the LAIPP, as a first line for treatment of FEP-SUD patients.

The most common side effects associated with LAIPP are injection site reactions, extrapyramidal symptoms, hyperprolactinemia, sedation, hypersalivation, orthostatic hypotension, tachycardia, and weight gain. Hypersensitivity reactions have rarely been reported and may be dose-dependent.

We report a case of a 20-year-old female, without medical history and no history of allergies, who was medicated with once-monthly LAIPP at dose 100 mg for the maintenance treatment of a first psychotic episode associated with cannabis abuse.

Approximately 24 hours after the first monthly injection dose, she was admitted in the emergency room (ER) presenting an increasing angioedema associated with stridor, requiring endotracheal intubation and administration of adrenaline, clemastine and hydrocortisone during the assessment in the ER.

After clinical stabilization, she was transferred to the internal medicine ward, and following a full recovery, she was discharged 6 days later while being medicated with Olanzapine 15 mg/day, Lorazepam 3 mg/day and Sertraline 50 mg/day. LAIPP was suspected as the etiology of the anaphylaxis reaction due to temporal relationship of its onset with therapy administration and by the exclusion of other potential causes. Consequently, LAIPP was discontinued at discharge.

**Conclusions:**

This report shows the possibility of a late and potentially life-threatening anaphylactic reaction to LAIPP. So, all physicians should be aware of this potential complication, which requires timely recognition and management.

**Disclosure of Interest:**

None Declared

